# The menstrual cycle regularity and skin: irregular menstrual cycle affects skin physiological properties and skin bacterial microbiome in urban Chinese women

**DOI:** 10.1186/s12905-023-02395-z

**Published:** 2023-05-31

**Authors:** Laiji Ma, Hong Jiang, Tingting Han, Yanqin Shi, Man Wang, Shanshan Jiang, Suzhen Yang, Lingyun Yao, Qingwen Jia, Li Shao

**Affiliations:** 1 School of Perfume and Aroma Technology, Shanghai Institute of Technology, Shanghai, 201418 China; 2 The Oriental Beauty Valley Research Institute, Shanghai Institute of Technology, Shanghai, 201403 China; 3 R&D Innovation Center, Shandong Freda Biotech Co., Ltd., Jinan, 250101 Shandong China; 4 Shanghai Jiaotong University Affiliated Sixth People’s Hospital, South Campus, Shanghai, 201499 China

**Keywords:** Menstrual cycle, Skin physiological parameters, Skin microbiota, Skin barrier, TEWL, Hormones

## Abstract

**Background:**

The regularity of the menstrual cycle directly affects women’s health. Many studies have focused on menstrual health; however, menstrual cycle regularity-related variations in skin physiological characteristics and skin microbiota have been seldom investigated.

**Methods:**

To investigate the menstrual cycle regularity-related variations in skin physiological characteristics and skin microbiota of 197 cases of Chinese women aged 18–35 years living in shanghai in 2021. Based on a self-evaluation questionnaire, the volunteers were divided into three groups C1 (those with a regular menstrual cycle), C2 (those with a less regular menstrual cycle) and C3 (those with an irregular menstrual cycle). The physiological parameters of facial skin were measured by non-invasive methods and the skin microbiome was analyzed by 16S rRNA high-throughput sequencing.

**Results:**

In the C3 group, the hydration content was significantly decreased (*p* < 0.05), the TEWL was significantly increased (*p* < 0.05), and the sebum content was increased (*p* > 0.05), indicating that the skin barrier integrity weakened with increased menstrual cycle irregularity. Additionally, the melanin level, *L* value and *b* value were significantly decreased (*p* < 0.05) in the C3 group, but the *a* value was significantly increased (*p* < 0.001), which indicated that the skin color became darker. Furthermore, the skin microbiota diversity decreased with increasing cycle irregularity, but the differences were not significant. The skin microbiota composition showed that the proportion of *Firmicutes, Acinetobacter, Staphylococcus* and *Cutibacterium* were increased in those with an irregular menstrual cycle, indicating that alterations in the ratio of bacterial phyla and/or genera might disturb skin homeostasis. Spearman correlation analysis revealed strong correlations between the microbiota and skin physiological parameters. Based on the associations among hormones, skin physiological parameters and skin microbiota, it is possible that the skin physiological parameters, as well as the skin microbial diversity and composition, change with hormonal fluctuations during the menstrual cycle.

**Conclusions:**

An irregular menstrual cycle can affect skin physiological characteristics and the skin microbiota. Female with an irregular menstrual cycle should strengthen skin care practices and use skin care products with moisturising and soothing effects to protect their skin.

## Introduction

The female menstrual cycle is characterized by natural fluctuations in oestrogen and progesterone, two ovarian gonadal hormones that rise and fall at predictable intervals [1, 2]. Previous studies have proven that menstrual cycle patterns strongly influence one’s psychosomatic state and physical well-being [3, 4]. A regular menstrual cycle is an important indicator of a healthy reproductive system and a woman’s health; however, many social and individual factors influence menstrual cycle regularity, such as health status, employment, stress, sleep patterns, diet, smoking, obesity and excessive weight loss [5–11]. Menstrual cycle irregularity aggravates the frequency and degree of menstrual symptoms [12]. Many adolescent and adult females experience menstrual problems, including skin diseases.

Skin physiological characteristics and several skin ailments are considered to be directly influenced by menstruation [13, 14]. Research has confirmed that physiological changes are associated with the menstrual cycle, and hormone fluctuations play important roles in regulating skin physiological parameters during the menstrual cycle [15]. Numerous characteristics of the epidermis, including skin surface lipid secretion and sebum production, skin thickness, skin hydration, barrier function, dermal collagen content, skin pigmentation, UV susceptibility and resident microflora can vary with the cyclically fluctuating levels of oestrogen and progesterone [13]. Oestrogen is thought to prevent skin aging in several ways, such as maintaining skin moisture by increasing the production of acidic mucopolysaccharides and hyaluronic acid in the skin and increasing the skin collagen content, which maintains skin thickness. Oestrogen may offer a valid therapeutic option for wrinkling by enhancing the morphology and synthesis of elastic fibres, collagen type III and hyaluronic acid [16]. The presence of various dermatoses correlates with peak levels of progesterone. For example, dermatoses that are perimenstrually exacerbated include acne, psoriasis, atopic eczema, irritant dermatitis and, possibly, erythema multiforme [13]. Therefore, hormone fluctuations and other factors that influence the menstrual cycle may strongly impact skin physiological properties [14]. Recent menstrual-related skin research has mainly focused on skin diseases, such as perimenstrually exacerbated dermatoses, while less attention has been given to the effects of mildly irregular cycles on the skin. The associations between menstrual cycle regularity and skin physiological characteristics are not yet clear.

The skin has a complex and dynamic ecosystem that is inhabited by many different microbial communities [17]. The skin microbiota plays an important role in the induction, education and function of the host immune system [18]. For example, dysbiosis of the microbiome can influence keratinocyte regulation and homeostasis, as well as the skin barrier function [19]. The importance of skin microbial communities has been increasingly recognized in recent years due to their influence on skin health and relation to various dermatological problems [20]. In healthy adults, the microbial composition primarily depends on the skin physiology and typically remains stable over time despite the skin’s continuous exposure to the environment [21]. However, the skin microbiome composition can be altered by internal and external factors such as inflammation and topically applied drugs [22, 23]. Alterations of the skin microbial composition may have important consequences for health and disease outcomes among individuals; indeed, some skin microbiota diversity patterns may be predictive or diagnostic of certain diseases [24]. In 2005, Farage et al. [14] reported that microbial counts fluctuated over the course of the menstrual cycle, indicating that the skin surface microflora was related to the menstrual cycle. Owing to the limited research methods at that time, precise changes in the skin microorganism composition were not identified. In the current study, we investigated whether menstrual cycle regularity affects skin physiological characteristics, including the skin microbiota.

The effects of physiological and lifestyle factors on skin physiological characteristics have been extensively investigated. For example, a previous study investigated the associations between skin biophysical parameters (including sebum production, hydration and pH) and age, sex and lifestyle factors among Chinese individuals [25, 26]. However, few studies have evaluated the associations between menstrual cycle regularity and skin physiological characteristics among urban Chinese women. In this work, we examined the associations between menstrual cycle regularity and skin physiological properties and skin microbiota among urban Chinese women. The results provide a better understanding of how skin biophysical properties and skin microbiota change throughout the menstrual cycle and provide skin care guidance for women with irregular menstruation.

## Materials and methods

### Participants and inclusion criteria

This study investigated the correlations between the skin health status and skin microbiota of qualified participants living in Shanghai (ages 18 ~ 60, *n* = 494). All participants provided written informed consent. This research was approved by the South Campus of the Sixth People’s Hospital Affiliated to Shanghai Jiaotong University (approval no.: 2021-KY-15) and abided by the ethical guidelines of the Declaration of Helsinki. The participants had no skin diseases, and in the 3 months before the study they had not taken any oestrogen, progesterone, corticosteroids or antibiotics. In addition, polycystic ovary syndrome, lactating and pregnant women were excluded. All participants filled out the self-evaluation questionnaire, which asked about their skin condition, lifestyle factors and physiology. All skin parameters and microbial samples were collected in July 2021, and skin testing was conducted during the non-menstruation phase.

A total of 262 female participants aged 18 ~ 60 years were initially enrolled in this study. Considering that the menstrual cycle is usually the most irregular at either end of the reproductive life (menophania and menopause) [27] and that skin physiological parameters and skin microbiota are significantly influenced by age and skin site [28], we finally selected a total of 197 healthy women aged 18 ~ 35 years as the research participants.

### Definition and grouping of “regular menstrual cycle”

**Fig. 1:**
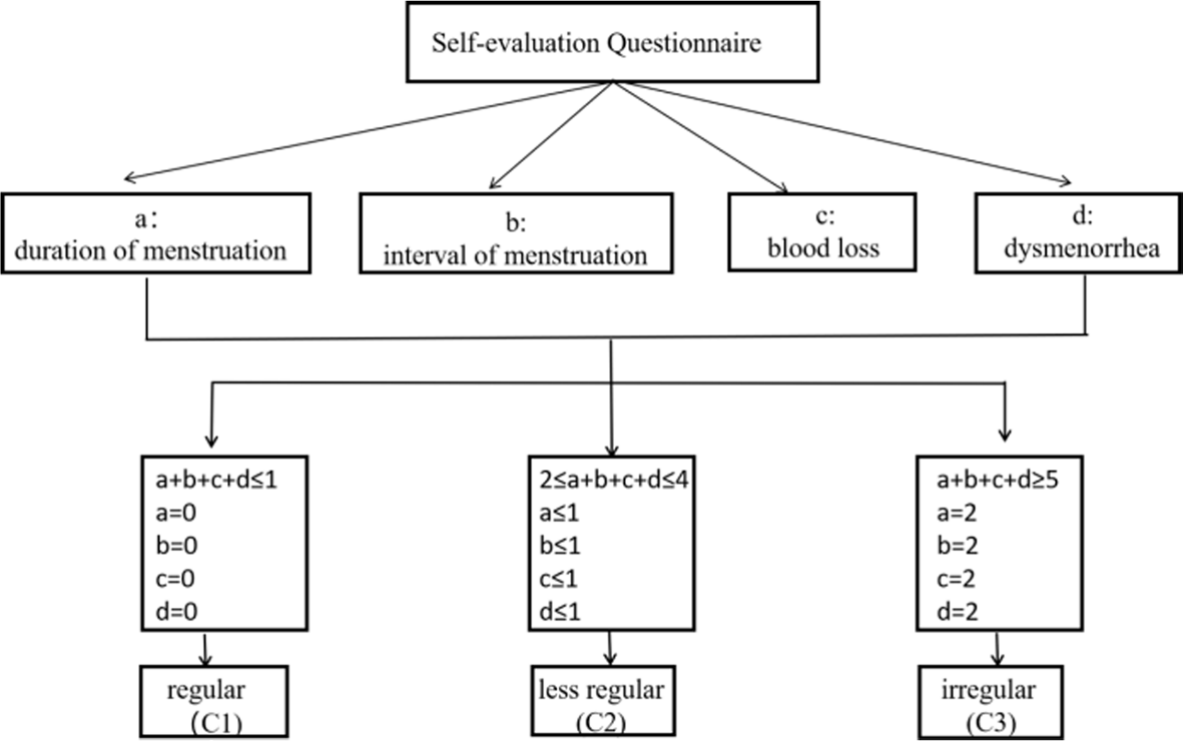
Customized scoring standard for “Regular cycles” (C1), “Less regular cycles” (C2) and “Irregular cycles” (C3) groups; if duration of menstruation/interval of menstruation/blood loss/dysmenorrhea gets is assessed as 2 points, the total score, even if only have 2 points, is still in the Irregular cycles group

In general, the menstrual regularity is evaluated from terms of “duration”, “interval between menstrual cycles”, “blood volume”, and “dysmenorrhea”. Therefore, in our current study, the duration of menstruation, interval of menstruation, blood loss and dysmenorrhea obtained from the questionnaire were used to evaluate the menstrual regularity. The grading standard is shown in Table 1 and the customized scoring standard was illustrated in Fig. 1. According to our customized scoring standard, the female participants were divided into three groups: C1 (regular menstrual cycle), C2 (less regular menstrual cycle) and C3 (irregular menstrual cycle). The grouping and basic information of each group was shown in Table 2.

**Table 1:**
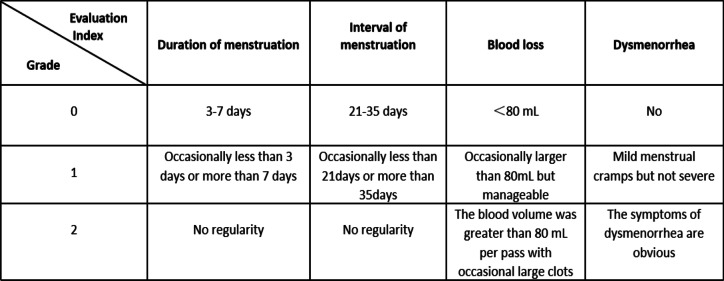
The grading standard of regular menstrual cycle

**Table 2:** General characteristics of the participants

Characteristics	C1(Regular cycles)	C2(Less regular cycles)	C3(Irregular cycles)	No. n (%)
Number participants, n (%)	49(24.9)	131(66.5)	17(8.6)	197
Age (yr), mean$\pm$SD	25.78 ± 4.52	25.65 ± 5.26	23.41 ± 5.14	25.72 ± 5.15
BMI(kg/m^2^), mean$\pm$SD	22.5 ± 5.12	22.4 ± 5.63	20.6 ± 4.13	21.8 ± 4.78
Skin changes throughout the menstrual cycle, n (%)				
Yes	36(73.5)	109(83.8)	10(58.8)	155(79.1)
No	13(26.5)	21(16.2)	7(41.2)	41(20.9)
Skin becomes worse before, during or after menstruation, n (%)				
1 week	32(65.3)	84(64.6)	8(47.1)	124(63.2)
before	8(16.3)	28(21.5)	3(17.6)	39(19.9)
1 week after	2(4.1)	7(5.4)	0	9(4.6)
No obvious changes in skin	7(14.3)	11(8.5)	6(35.3)	24(12.2)
Characteristics of worsened skin condition, n (%)				
Dry	11(22.4)	20(15.4)	1(5.8)	32(16.3)
Gloomy	22(44.9)	48(36.9)	3(17.6)	73(37.2)
Pigmentation	6(12.2)	11(8.5)	2(11.7)	19(9.7)
Prone to acne	22(44.9)	59(45.4)	9(52.9)	90(45.9)
Increased sebum secretion	12(24.5)	40(30.8)	3(17.6)	55(28.1)
Decreased sebum secretion	2(4.1)	2(1.5)	0	4(2.0)
Other	2(4.1)	11(8.5)	3(17.6)	16(8.2)
No obvious changes in skin	4(8.1)	11(8.5)	4(23.5)	19(9.7)
Treat body/skin before, during or after menstruation, n (%)				
Yes	9(18.4)	17(13.1)	0	26(13.3)
No	40(81.6)	113(86.9)	17(100)	190(96.9)

No significant differences in Age and BMI among three groups

### Skin physiological parameter collection

All participants were required to not use skin care products or cosmetics after washing their face the night before the measurement and the day of the test. Before the test, the participants rested for at least 30 min in an indoor environment controlled at 20 ~ 22 °C with a relative humidity of 40% ~ 60%. The following skin physiological parameters of the cheek regions were measured with different instruments from Courage + Khazaka electronic GmbH, Germany: skin hydration in the stratum corneum (Corneometer CM 825), trans-epidermal water loss (TEWL) (Tewameter TM 300), skin sebum (Sebumeter SM 815), skin pH (Skin-pH-Meter pH 905), skin gloss (Glossymeter GL200), skin melanin and haemoglobin content (Mexameter MX 18), skin wrinkles and smoothness (Visioscan VC 98), skin elasticity (R2) and firmness (F4) (Cutometer dual MPA580). The *L*, *a* and *b* values were also measured (VISIA-CR system, Canfield, USA). The physiological parameter results are provided in Table 3.

**Table 3:** Skin physiological parameters in three participant groups

Physiological parameter	Group		
	**C1(Regular cycles)**	**C2(Less regular cycles)**	**C3(Irregular cycles)**
Hydration^**^	70.5853 ± 9.48	65.0591 ± 12.58^a**^	62.2106 ± 12.18^b*^
TEWL^*^	21.018 ± 3.88	21.373 ± 4.233^c*^	24.053 ± 4.841^b*^
Sebum	70.31 ± 39.328	88.27 ± 56.840	79.59 ± 46.016
Gloss	9.4396 ± 2.4548	9.2459 ± 2.626	9.3635 ± 2.425
Melanin^*^	155.78 ± 31.143	143.915 ± 28.205^a*^	150.741 ± 24.996
Haemoglobin	323.64 ± 60.001	339.671 ± 58.028	357.847 ± 48.932
Elasticity(R2)	0.799 ± 0.063	0.778 ± 0.064	0.781 ± 0.069
Firmness(F4)	15.439 ± 4.774	14.762 ± 4.410	16.046 ± 3.477
pH	5.042 ± 0.407	5.124 ± 0.415	5.120 ± 0.446
L^*^	62.4757 ± 3.523	60.6415 ± 2.466^a**^	60.4214 ± 1.215^b*^
a^***^	13.8943 ± 3.188	15.3766 ± 2.071^a***^	16.4188 ± 1.534^b***^
b^**^	14.620 ± 1.671	13.9819 ± 1.821^a*^	13.0847 ± 1.236^b**,c*^
Wrinkle	88.9797 ± 44.541	89.9689 ± 29.513	94.5208 ± 29.632
Smoothness^*^	237.75 ± 77.842	207.6715 ± 58.771^a*^	200.9626 ± 69.144

*L* represents brightness, with the darkest black indicated by *L* = 0 and the brightest white indicated by *L* = 100. *a* represents the green–red component, with green in the negative direction and red in the positive direction. *b* represents the blue-yellow component, with blue in the negative direction and yellow in the positive direction. C1: Group with regular menstrual cycles; C2: Group with less regular menstrual cycles; C3: Group with irregular menstrual cycles. Difference test method: Wilcoxon rank–sum test, a: differences test between C1 and C2 groups; b: differences test between C1 and C3 groups; c: differences test between C2 and C3 groups

^*^p < 0.05

^**^p < 0.01

^***^p < 0.001

### Skin microbial specimen collection

All participants were instructed to not wash their face the night before and to collect skin bacteria the next morning. The skin microbial specimens were collected in accordance with a previously reported method [26]. Briefly, the samples were obtained from a 3 cm^2^ area of the cheeks by swabbing with a sterile wetting solution (0.9% NaCl and 0.1% Tween-20). The sampling regions were swabbed approximately 30 times for at least 20 s in total. After collection, the samples were immediately stored at − 80 °C for subsequent DNA extraction.

### DNA extraction and sequence analysis

DNA was extracted from the skin microbial samples using the commercial FastDNA Spin Kit (MP Biomedicals, USA) following the instructions provided by the manufacturer. The V3-V4 variable region in the 16S rRNA gene was PCR-amplified with the primers 338F (5′-ACTCCTACGGGAGGCAGCAG-3′) and 806R (5′-GGACTACHVGGGTWTCTAAT-3′) using a thermocycler (GeneAmp 9700, ABI). PCR amplicons were purified using the AxyPrep DNA Gel Extraction Kit (Axygen Biosciences, USA), libraries were prepared using the NEXTFLEX Rapid DNA-Seq Kit (Bioo Scientific, USA), and sequencing was performed using the Illumina MiSeq 300-bp paired-reads platform (Illumina, USA) in accordance with standard protocols provided by Majorbio Bio-Pharm Technology Co. Ltd. (Shanghai, China) [29].

### Processing the sequencing data

The sequencing data were processed using a previously reported method with some modifications [30]. The 16S rRNA gene sequencing reads were demultiplexed and quality-filtered by FASTP (https://github.com/OpenGene/fastp, version 0.20.0) and merged by FLASH (http://www.cbcb.umd.edu/software/flash, version 1.2.7). The operational taxonomic units (OTUs) were clustered by applying a 97% similarity cut-off using UPARSE (http://drive5.com/uparse/, version 7.1), and chimeric sequences were identified and removed using UCHIME. The taxonomy of each OTU representative 16S rRNA gene sequence was classified by the RDP classifier algorithm (http://rdp.cme.msu.edu/) by referencing the SILVA database (V138) and applying a confidence threshold of 70%.

The raw data from high-throughput sequencing were collated and filtered, and validated sequences were obtained for subsequent analysis. Approximately 5,127,549 valid sequences were obtained. All samples were flattened according to the minimum sequence number. A total of 12,225 OTUs were obtained, belonging to 60 phyla, 1,895 genera and 4,136 species.

### Statistics analysis

All data are represented as the mean ± standard deviation (X ± SD) unless otherwise indicated. The statistical significance level was 0.05 unless otherwise noted. A nonparametric test and the Kruskal–Wallis test were used to compare the skin physiological properties among the tested groups. SPSS version 25.0 (IBM Corp., USA) was used for the statistical analyses. For the microbial diversity analysis, the Bray–Curtis distance was used in the principal coordinate analysis, and the non-parametric Wilcoxon rank–sum test was used to compare the microbial diversity and composition between two groups. The data were analyzed on the online Majorbio I-Sanger Cloud Platform (www.i-sanger.com).

## Results

### General characteristics of the participants

The menstrual cycle regularity of the female participants and menstrual cycle-associated skin characteristics were obtained from the self-evaluation questionnaire. Nearly 9% of the participants reported an irregular menstrual cycle, and 66.5% reported a less regular menstrual cycle (Table 2). Thus, most of the women reported a less regular menstrual cycle. In this study, we examined the impacts of menstrual cycle regularity on the skin status. Approximately 80% of women reported changes in their skin characteristics throughout the menstrual cycle, and the most obvious changes occurred 1 week before menstruation, which was consistent with previous studies [31, 32]. The reported changes in skin physiological parameters affected by the menstrual cycle included the skin becoming dry and darker, increased sebum secretion, and the appearance of acne; these changes were reported by a large proportion of participants in all groups. Overall, 96.9% of the participants made no changes to their body or skin care routine during or after menstruation.

### Irregular menstrual cycle affected the skin physiological properties

The skin physiological properties of the three tested groups (C1, C2 and C3) are summarized in Table 3. Compared with C1 (those with a regular menstrual cycle), C2 (those with a less regular menstrual cycle) and C3 (those with an irregular menstrual cycle) had significantly decreased (*p* < 0.05) hydration, melanin, *L*, *b* and smoothness values (Table 3). The skin gloss and elasticity (R2) parameters were also decreased (*p* > 0.05) in the C2 and C3 groups. The less regular and irregular menstrual cycles among the C2 and C3 groups may have been caused by irregular hormone fluctuations, which might have resulted in the decreased skin hydration and melanin content (Table 3).

Compared with the C1 group, the C2 and C3 groups had significantly increased TEWL and *a* values (*p* < 0.05); the sebum content was also increased (*p* > 0.05) (Table 3). In addition, the haemoglobin value was significantly increased (*p* = 0.037) in the C3 group but only moderately increased (*p* > 0.05) in the C2 group. Skin wrinkles were moderately increased in the C2 and C3 groups (*p* > 0.05) compared to C1. There were minor differences in skin pH (*p* > 0.05) among the three tested groups. The sebum content of the skin is related to the menstrual cycle because oestrogens regulate the production of sebum and other stratum corneum lipids [14]. The higher sebum contents in the C2 and C3 groups (Table 3) may have been influenced by oestrogen production and the skin microbial composition [1, 14].

### Alpha and beta diversity analysis

**Fig. 2:**
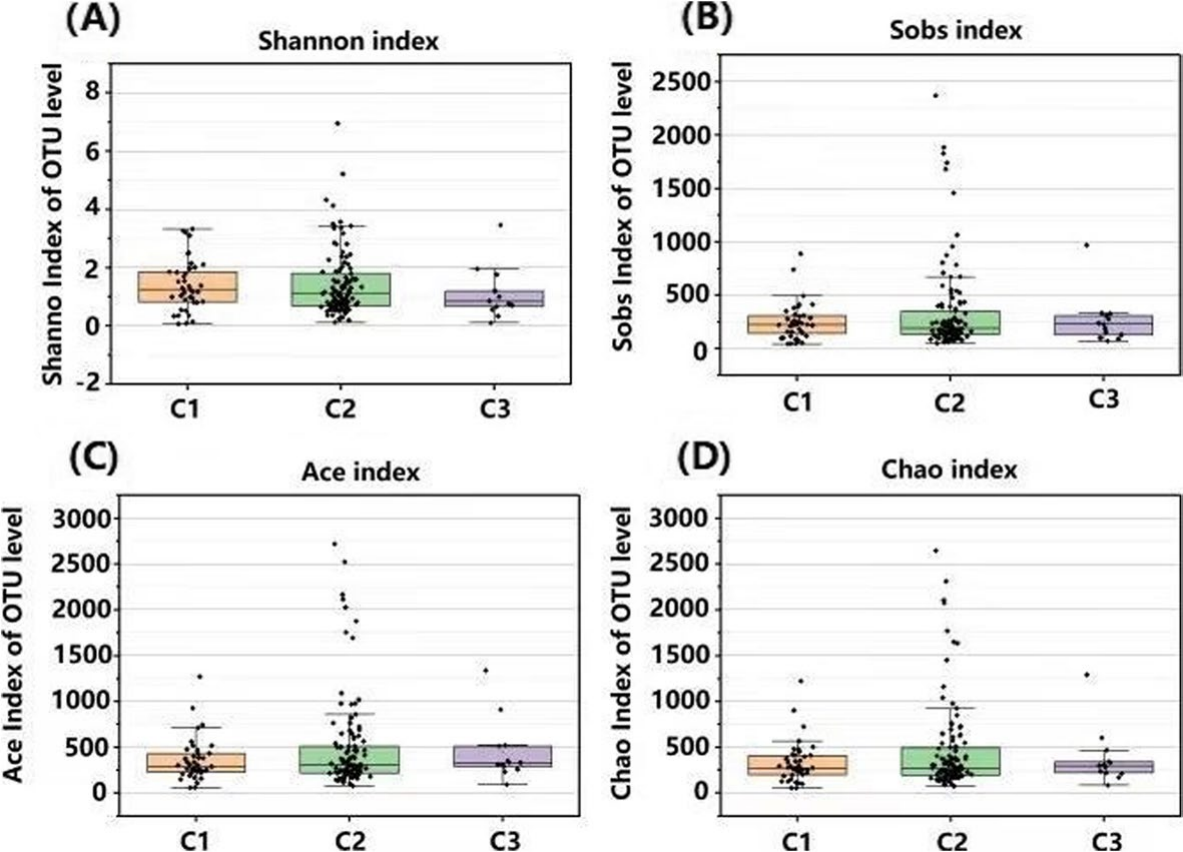
Bacterial alpha diversity of the three groups. **A** Shannon index; **B** Sobs index; **C** Ace index; **D** Chao index. C1: Group with a regular menstrual cycle; C2: Group with a less regular menstrual cycle; C3: Group with an irregular menstrual cycle

The alpha diversity of the skin microbiome in the three groups was analysed based on the obtained Sobs, Ace and Chao richness values and the Shannon diversity values (Fig. 2A-D). The Shannon diversity index decreased as the menstrual cycle irregularity increased (from C1 to C3) (*p* > 0.05) (Fig. 2A). The Sobs, Chao and Ace indexes of the C3 group were slightly increased (*p* > 0.05) compared to C1 (Fig. 2B-D). In the C2 group (those with a less regular menstrual cycle), the observed Sobs value was higher (*p* > 0.05) than in C1 but lower (*p* > 0.05) than in C3. These results indicated that the skin microbiomes of the C3 group (those with an irregular menstrual cycle) had poorer species abundance. As menstrual cycle irregularity increased, the skin microbiota diversity decreased, although no significant (*p* > 0.05) differences were observed among the three groups.

The beta diversity (inter-sample) analysis revealed no significant (*p* > 0.05) differences among the three tested groups (data not shown), suggesting a high similarity in the microbial composition. As previously reported, the stability of a healthy human skin microbiome is largely skin-site specific [33], and significant changes in microbial diversity are probably a hallmark of a disease state [34]. In this work, healthy participants were divided into three groups (C1, C2 and C3) according to their menstrual cycle regularity (Table 2). No significant variations in beta diversity were observed among the three groups, probably because healthy adults maintain a stable skin microbiome [35].

### Taxonomic profiles of skin bacteria in the three groups

**Fig. 3:**
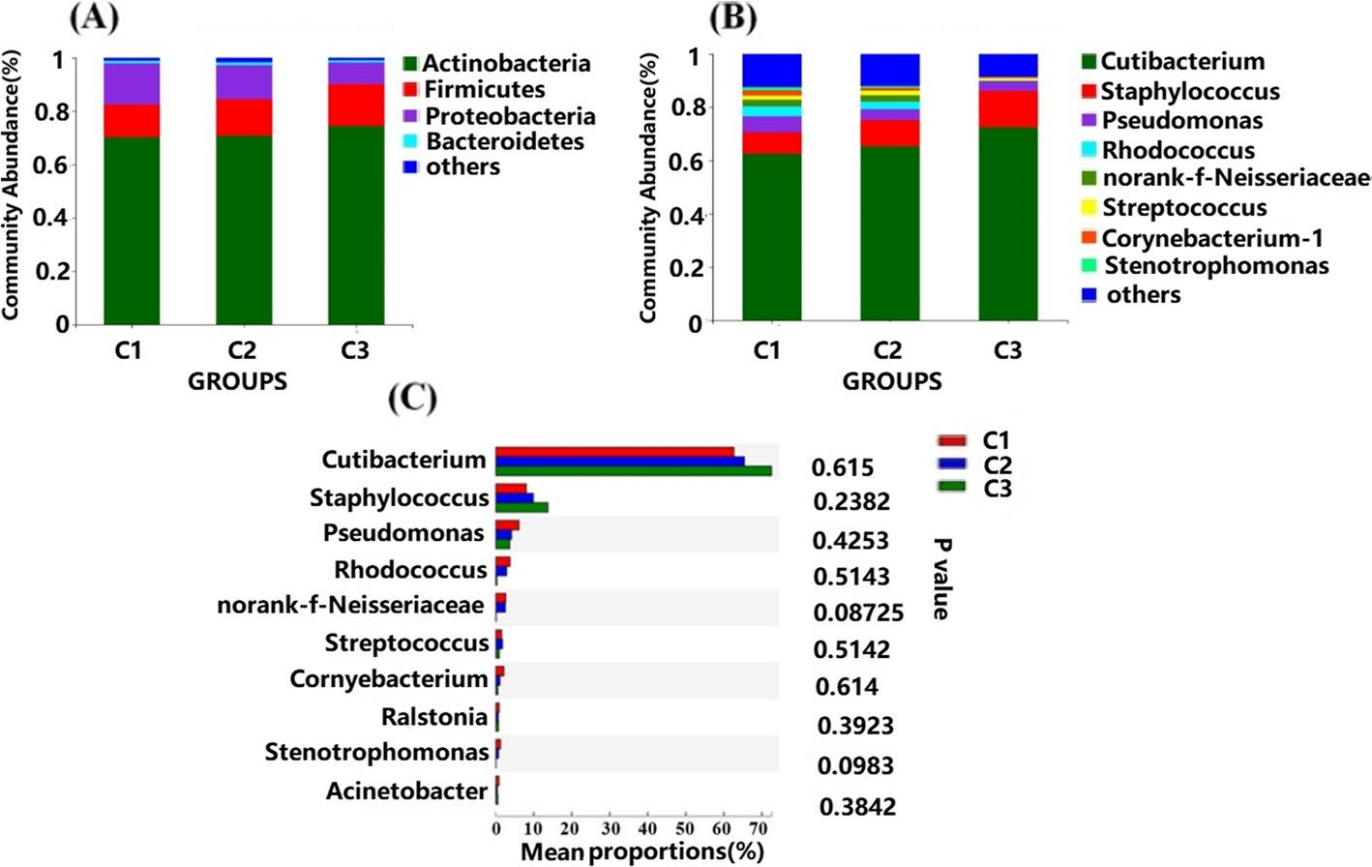
Bacterial composition analysis of groups C1, C2 and C3. **A** Phylum-level species abundance; **B** Genus-level species abundance; **C** Differences in genus-level species abundance; C1: Group with a regular menstrual cycle; C2: Group with a less regular menstrual cycle; C3: Group with an irregular menstrual cycle. Differences test method: Wilcoxon rank–sum test

The dominant microbial composition in all groups was *Actinobacteria* (> 70%), *Firmicutes* (> 12%), *Proteobacteria* (> 8%) and *Bacteroidetes* (< 2%) based on the phylum level, accounting for more than 98% of the bacteria (Fig. 3A) [29]. As shown in Fig. 3A, increased menstrual cycle irregularity was associated with an increased abundance of *Acinetobacter* and *Firmicutes* (*p* > 0.05), but the abundance of *Proteobacteria* decreased as irregularity increased (*p* > 0.05). According to a recent report on the skin microbiome, shifts in the bacteria phyla/genera are disease status-dependent [35], Our results showed that there were some differences in the proportions of bacterial phyla among the groups, but these differences were not significant (Fig. 3A). For example, the combined proportion of *Proteobacteria* and *Acinetobacter* was lower in C3 (10.78%) than in C1 (21.87%). Additionally, the combined proportion of *Firmicutes* and *Acinetobacter* was higher in C3 (21.15%) than in C1 (17.43%). Thus, the impact of shifts in the proportion of phyla on skin health and disease should be further investigated.

At the genus level, the skin bacteria in the three groups were mainly composed of *Cutibacterium*, *Staphylococcus*, *Pseudomonas*, norank_f_*Neisseriaceae*, *Rhodococcus*, *Streptococcus*, *Corynebacterium*, *Ralstonia* and *Stenotrophomonas* (Fig. 3B-C). With increasing menstrual cycle irregularity, the *Cutibacterium* and *Staphylococcus* abundance increased (*p* > 0.05), while the *Pseudomonas*, *Rhodococcus* and *Corynebacterium* abundance decreased (*p* > 0.05), and the abundance of *Stenotrophomonas* significantly decreased (*p* < 0.05). Similar to previous results, *Cutibacterium* and *Staphylococcus* were the main facial microbiota genera [29], and the ratio of *Staphylococcus* to *Cutibacterium* increased with increasing menstrual cycle irregularity. By contrast, the ratios of *Pseudomonas* to *Cutibacterium* and *Rhodococcus* to *Cutibacterium* were decreased. These results suggested that there were differences in the skin microbiome at the genus level among the three groups.

### Correlation between skin bacteria and skin physiological parameters

**Fig. 4:**
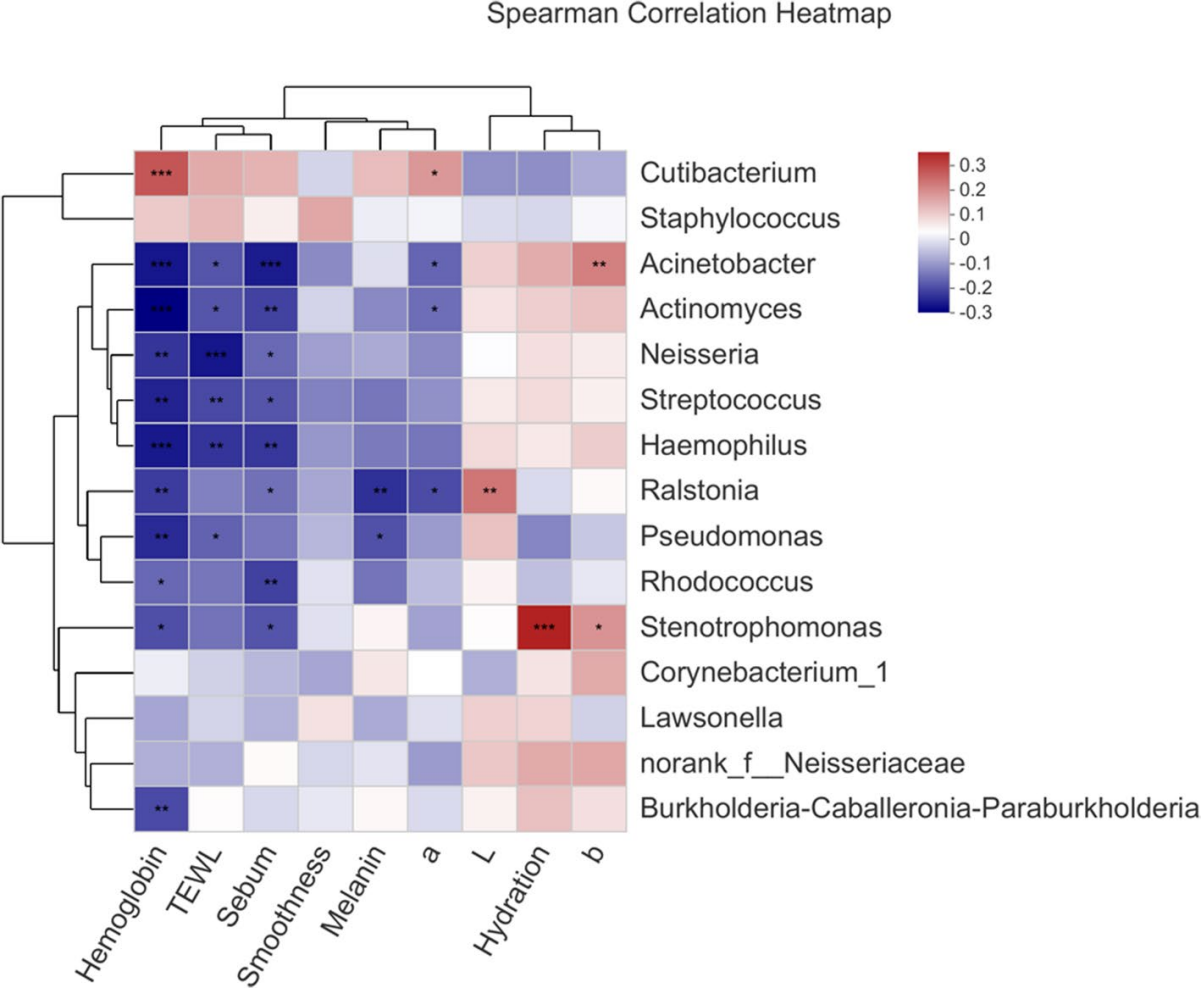
Spearman correlations between facial bacterial abundance and skin physiological parameters. Only the top 15 most-abundant genera are shown; (*p < 0.05, **p < 0.01, ***p < 0.001)

Spearman correlations between skin physiological parameters (sebum, hydration, TEWL, haemoglobin, melanin, *L*, *a*, *b* and smoothness) and the skin bacteria were analysed. The correlation heatmap showed that the relationships between bacterial genera and skin physiological parameters differed (Fig. 4). The relative abundance of *Cutibacterium* was positively correlated with the sebum content (*r* = 0.128,* p* > 0.05), haemoglobin content (*r* = 0.259, *p* < 0.001), *a* value (*r* = 0.1656, *p* < 0.05), TEWL (*r* = 0.140,* p* > 0.05) and melanin content (*r* = 0.115, *p* > 0.05). The positive correlation between the sebum content and *Cutibacterium* was consistent with the observation that these parameters were both increased in the C2 (less regular menstrual cycle) and C3 (irregular menstrual cycle) groups. *Stenotrophomonas* was significantly positively correlated with the skin hydration content (*r* = 0.331,* p* < 0.001) and* b* value (*r* = 0.173, *p* < 0.05) but negatively correlated with the haemoglobin content (*r* =  − 0.193, *p* < 0.05), sebum content (*r* =  − 0.187, *p* < 0.01) and TEWL (*r* =  − 0.150, *p* > 0.05). *Acinetobacter* was significantly positively correlated with the skin *b* value (*r* = 0.200, *p* < 0.01) but negatively correlated with the haemoglobin content (*r* =  − 0.263, *p* < 0.001), sebum content (*r* =  − 0.255, *p* < 0.001), TEWL (*r* =  − 0.186, *p* < 0.001) and *a* value (*r* =  − 0.166, *p* < 0.05). As shown in Fig. 4, the TEWL, haemoglobin and sebum contents were significantly negatively correlated with *Pseudomonas*, *Rhodococcus*, *Streptococcus*, *Ralstonia, Neisseria*, *Actinomyces, Stenotrophomonas* and *Haemophilus*; these bacteria were positively correlated with skin hydration. Similar relationships between the skin bacterial community and other skin parameters have been previously reported. Zheng et al. reported that *Streptococcus* and *Actinomyces* had a significant negative correlation with sebum secretion [29]. Our results and those of previous studies indicate that the composition and abundance of skin bacteria are closely related to skin physiological parameters.

## Discussion

Menstrual health is an integral part of overall health for females, and most women menstruate between menarche and menopause [36]. However, many women experience menstrual cycle irregularity, which can have a significant impact on their physical, mental and social well-being [37]. Menstrual cycle regularity is reportedly influenced by a variety of physical and mental conditions and health-related lifestyle factors. In our investigation, most participants reported some level of menstrual cycle irregularity. Menstrual cycle irregularity has been associated with serious health outcomes. However, most menstrual-related skin research has focused on skin diseases, while less attention has been given to the effect of mild cycle irregularity on the skin. This work was the first investigation of the association between menstrual cycle regularity and skin physiological characteristics and skin microbiota among urban Chinese women.

As menstrual cycle irregularity increased (from C1 to C3), the skin hydration content significantly decreased (*p* < 0.05), the TEWL significantly increased (*p* < 0.05) and the sebum content increased (*p* > 0.05). The skin hydration content, TEWL and sebum content are considered relevant indicators that reflect the skin barrier function [38–40]. The skin physiological parameters indicated that the skin barrier integrity weakened as menstrual cycle irregularity increased. Skin physiological changes are associated with the menstrual cycle [14]. The cyclically fluctuating levels of oestrogen and progesterone influence skin hydration, the sebum content and TEWL. Oestrogens can improve the water-binding capacity of the stratum corneum and dermis, and they suppress the production of sebum and other stratum corneum lipids in the skin [14]. Therefore, we speculate that oestrogen levels were relatively decreased and androgen levels were relatively increased in the irregular menstrual cycle group (C3). The data from the questionnaire about skin characteristics (e.g. skin dryness, sebum production and presence of acne) indicated that the skin physiology changed as the hydration content decreased and the sebum content increased. Previous studies have shown that among those with irregular menstrual cycles, the frequency and degree of menstrual symptoms, such as the premenstrual exacerbation of acne, atopic dermatitis, and oestrogen dermatitis, are increased [13].

The key parameters of skin color and pigmentation, which are the melanin level,* L* value, *a* value and *b* value, revealed significant changes with increasing menstrual cycle irregularity. Compared with the regular menstrual cycle group, in the irregular menstrual cycle group, the melanin level, *L* value and *b* value were significantly decreased (*p* < 0.05) and the *a* value was significantly increased (*p* < 0.001). These results indicated that the skin color was darker in the C3 group. Significant changes in the melanin, *L*, *a* and *b* values indicated that the skin color and pigmentation were affected by an irregular menstrual cycle. Previous studies have attributed variations in skin pigmentation to fluctuations in oestrogen, as this hormone can stimulate epidermal melanogenesis [41]. Oestrogen can stimulate epidermal melanogenesis during certain stages of the menstrual cycle; it can increase skin pigmentation in the luteal phase, and sometimes also in the menstrual phase [14, 41]. We speculate that the oestrogen levels fluctuated widely in the irregular menstrual cycle group, but that they were lower than in the regular menstrual cycle group.

Interestingly, the microbial diversity and microbial composition of the cheek regions changed with increasing menstrual cycle irregularity. As shown in Fig. 2, the skin microbial diversity (the Shannon index) was decreased in the C3 group, although the differences among groups were not significant. The skin microbiota composition showed that the ratios of *Firmicutes*, *Acinetobacter* and *Staphylococcus* to *Cutibacterium* increased, while the ratio of *Proteobacteria* to *Acinetobacter* decreased with increasing menstrual cycle irregularity, although the differences among groups were not significant (*p* > 0.05) (Fig. 3). The skin microbiome composition is highly dependent on the skin microenvironment [21]. With increased menstrual cycle irregularity, the skin hydration significantly decreased and the sebum content increased; therefore, the skin was more suitable for the growth of oil-loving microorganisms [42]. We observed that the abundances of *Cutibacterium* and *Staphylococcus* increased and the alpha diversity of the skin decreased accordingly. A similar conclusion was obtained from a study of skin types and the diversity of skin flora; in that study, the sebum content was negatively correlated with the alpha diversity index [29]. *Cutibacterium acne* and *Staphylococcus epidermidis* are the main bacterial species on facial skin and are known as sentinel bacteria [43]. The relative abundance of *C. acne* affects skin health and plays a crucial role in maintaining homeostasis [44]. The ratio of *Staphylococcus* to *Cutibacterium* was increased with increasing menstrual cycle irregularity, indicating that a shift in the ratio of bacterial phyla and/or genera might disturb skin homeostasis [35]. Hormones regulate skin physiological parameters such as sebum production and skin hydration, which affect the microbial diversity and composition; thus, the skin microflora might be correlated with hormonal changes. Our results and those of previous studies indicate that the microbial counts [14], as well as the microbial diversity and composition, can change with hormone fluctuations during the menstrual cycle.

The Spearman correlation analysis revealed close correlations between the microbiota genera and skin physiological parameters. The abundance of *Cutibacterium* was positively correlated with sebum, haemoglobin and the *a* value. In the cheek region, the C3 group showed a higher sebum content, a significantly decreased hydration content and a higher TEWL value (Table 3). The high abundances of *Cutibacterium* and *Staphylococcus* were correlated with a high TEWL value and low hydration value. Furthermore, the high abundance of *Cutibacterium* was correlated with high sebum secretion (Fig. 4). These data are in agreement with those of a previous report that found that oily skin was accompanied by high sebum secretion, a high TEWL value and a high *Cutibacterium* abundance*.* The TEWL, haemoglobin and sebum parameters had significant negative correlations with *Pseudomonas*, *Rhodococcus*, *Streptococcus*, *Ralstonia, Neisseria*, *Actinomyces, Stenotrophomonas* and *Haemophilus*, and these bacterial genera were positively correlated with skin hydration. These data further confirmed that the sebum content and skin hydration were significantly predictive of skin bacteria [42, 45].

The present study has some limitations. Menstrual cycle regularity was self-reported and therefore may have depended on subjective perceptions. Furthermore, biomedical markers and hormone levels were not measured. We focused on the impact of menstrual cycle regularity on the skin and did not investigate the causes of menstrual cycle irregularity or other symptoms associated with irregularity. Considering additional menstrual cycle-related factors in a future study may reduce the impact of variation in the results and conclusions.

## Conclusion

The results of this study indicate that menstrual cycle regularity can affect the skin physiology and microbiota. Compared with the regular menstrual cycle group, the irregular menstrual cycle group had a lower skin hydration content, a higher TEWL and sebum content and more pigmentation. They also had less microbial diversity and a different species composition. Irregular menstrual cycles are regulated by hormone fluctuations. These affected the normal function of the skin and caused skin health problems, such as premenstrual acne and eczema. Our data and those of previous studies indicate that an irregular menstrual cycle is associated with serious health problems such as breast cancer, diabetes, obesity and dermatoses. Therefore, those with irregular menstrual cycles should receive suitable treatment, particularly in serious cases, which may require medication. These individuals should also engage in healthy lifestyle practices such as eating a healthy diet, maintaining a reasonable weight and reducing stress. Moreover, our results suggest maintaining healthy skin care practices and using skin care products with moisturising and soothing effects to protect the skin in cases of an irregular menstrual cycle.

## Data Availability

The sequence dataset has been deposited in the National Center for Biotechnology Information Sequence Reads Archive Database (accession number: SRP332768).
